# Lymphatic Metastasis of NSCLC Involves Chemotaxis Effects of Lymphatic Endothelial Cells through the CCR7–CCL21 Axis Modulated by TNF-α

**DOI:** 10.3390/genes11111309

**Published:** 2020-11-04

**Authors:** Shuai Zhang, Hongzheng Wang, Zhiyun Xu, Yongkang Bai, Lin Xu

**Affiliations:** 1 Jiangsu Cancer Hospital & Jiangsu Institute of Cancer Research & The Affiliated Cancer Hospital of Nanjing Medical University, Nanjing 210009, China; xuezhongdaoke@163.com (S.Z.); zhiyunxu2012@gmail.com (Z.X.); 13057523980@163.com (Y.B.); 2 State Key Laboratory of Natural Medicines, Jiangsu Key Laboratory of Carcinogenesis and Intervention, Key Laboratory of Drug Quality Control and Pharmacovigilance, Ministry of Education, Jiangsu Key Laboratory of Drug Design and Optimization, China Pharmaceutical University, Nanjing 210009, China; whz605669671@163.com

**Keywords:** CCR7, CCL21, lymphatic metastasis, TNF-α, NSCLC

## Abstract

Metastasis and recurrence are the main causes of lung adenocarcinoma patients’ death. Lymphatic metastasis is the main way of non-small cell lung cancer (NSCLC) metastasis. C-C chemokine receptor type 7 (CCR7) overexpression has been demonstrated to mediate occurrence and progression of NSCLC. Moreover, Chemokine ligand 21 (CCL21) was used to activate CCR7. The CCR7–CCL21 axis is one of the most common “chemokine-receptor” modes of action in the development and metastasis of multiple tumors. However, the role of the CCR7–CCL21 axis in lymphatic metastasis of NSCLC is poorly understood. The study was conducted to investigate the molecular mechanism underlying CCR7–CCL21 axis-mediated lymphatic metastasis of NSCLC A549 cells. Tumor necrosis factor α (TNF-α) could regulate the tumor microenvironment balance by promoting chemokine secretion. Our study demonstrated that TNF-α promoted CCL21 production in human lymphatic endothelial cells (HLEC). Results further showed that TNF-α significantly activated the NF-κB pathway in HLEC. NF–κB pathway inhibition with ammonium pyrrolidinedithiocarbamate (PDTC) caused a significant decrease in CCL21 secretion, suggesting that TNF-α-induced CCL21 secretion in HLEC was through NF–κB pathway. Co-culture of A549 cells and TNF-α-treated HLEC confirmed that the metastasis of A549 cells was enhanced, meanwhile, apoptosis-related proteins were hardly affected. The data proved that a co-culture system prevented cell apoptosis while inducing the lymphatic metastasis of A549 cells. However, the situation was reversed after neutralizing CCL21 expression, suggesting that TNF-α-induced CCL21 secretion in HLEC is involved in A549 cells metastasis. Collectively, our finding demonstrated that NF-κB pathway-controlled CCL21 secretion of HLEC contributing to the lymphatic metastasis of A549 cells via the CCR7–CCL21 axis, validating the CCR7–CCL21 axis as a potential target to inhibit metastasis of NSCLC.

## 1. Introduction

Lung cancer is a malignant tumor with high morbidity and mortality worldwide, and its incidence is increasing year by year. About 80 percent of these lung cancers are non-small cell lung cancer (NSCLC) [1]. NSCLC is a type of epithelial lung cancer and is mainly divided into lung adenocarcinoma and lung squamous cell carcinoma [2]. NSCLC cells generally have specific mutations in DNA, which also give tumor cells some new characteristics [3]. At present, the treatment of lung cancer mainly includes chemotherapy, radiotherapy, surgical resection, targeted therapy and immunotherapy. Chemotherapy and radiotherapy either synchronously or alternately have a great effect in both early and late stages of small cell lung cancer, and can even cure early small cell lung cancer, but for NSCLC there is only about a 50% remission rate, nor can it cure NSCLC [4,5]. Resection is the main and the first choice for lung cancer treatment, and complete resection in NSCLC patients could achieve 40–70% 5-year overall survival. In addition, chemotherapy administered after complete resection improves overall survival at 5 years by approximately 5% [6]. Resection can completely remove the primary lung cancer and remove metastatic lymph nodes around the tumor, and also remove most of the tumor, which is beneficial for other treatments [7]. Despite recent advances in therapeutic regimens, the incidence and mortality rate of NSCLC are still very high [8]. However, lung cancer cells have a strong propensity to metastasize, which is an important reason for the high death rate of lung cancer. Most patients have metastasized at the first consultation, which has become an important factor affecting the prognosis of patients with NSCLC. Although comprehensive treatments have been adopted, the results have not yet been satisfactory. Metastasis and recurrence are the main causes of death of patients with NSCLC. Metastasis of lung cancer cells mainly includes lymphatic metastasis, blood vessel metastasis and tracheal metastasis [9], among which lymphatic metastasis is the main way of metastasis of NSCLC [10]. Blocking lymphatic metastasis of NSCLC has important significance for improving the prognosis of patients [11]. However, the intervention and treatment measures for cancer cells in the process of metastasis have not been paid enough attention. The reason is still that the underlying mechanism of lymphocytic metastasis in NSCLC cells is still largely obscure. More and more studies have shown that lymphatic metastasis plays an important role in the dissemination of tumor cells. Therefore, studies concerning the mechanism underlying the lymphatic vessel metastasis of NSCLC may have a particularly important impact on the systematic treatment of lung cancer.

Several cytokines in the microenvironment could assist lung cancer cells in invading and metastasizing. Among these cytokines, tumor necrosis factor (TNF-α) is common in the microenvironment of human lymphatic endothelial cells (HLEC). Chemokine ligand 21 (CCL21) is a chemokine highly expressed in lymph nodes and lymphatic endothelium [12]. Studies have shown that C-C chemokine receptor type 7 (CCR7), a G protein-coupled receptor, is the receptor of CCL21 and aberrantly expressed in certain tumor types. In addition, the high expression level of CCR7 has been identified in a wide variety of cancer cells, including breast cancer cells, rectal cancer cells, prostate cancer cells, esophageal squamous cell cells, and gastric cancer cells [13]. As a member of the C-C-like chemokine receptor family, CCR7 participates in the regulation of tumor cells including tumorigenesis, proliferation, invasion, and metastasis [14,15]. CCL21 is one of only two ligands for CCR7, the other one is Chemokine ligand 19 (CCL19). The mutual binding between CCL21 and CCR7 plays an important role in lymphatic metastasis of various tumors. Lymphatic metastasis is an early feature of lung cancer metastasis, and studies have shown that lymphatic metastasis is the main factor affecting the prognosis of lung cancer patients [16]. The present study was conducted to investigate the molecular mechanism underlying the CCR7–CCL21 axis-mediated lymphatic metastasis of NSCLC A549 cells.

NF-κB is a nuclear transcription factor and can regulate the expression of chemokines, inflammatory cytokines and immune-related receptors [17,18]. In the classical NF-κB pathway, cells are stimulated to activate the IκB protein kinase known as IKK, and the activated IKK can phosphorylate the IκB protein, leading to its ubiquitination and release of the dimer of NF-κB, which can then enter the nucleus and bind to the target gene to affect the transcription of target gene [19]. Activation of the NF-κB signaling pathway can regulate the expression of various proteins and cytokines associated with tumor invasion and metastasis, including matrix metalloproteinases, adhesion molecules, and urokinase-type plasminogen activators [20,21]. It is well known that TNF-α could significantly activate the NF-κB signaling pathway [22]. Therefore, it is worth studying whether the increased lymphatic metastasis in NSCLC with high expression of CCR7 is caused by CCL21 induced by TNF-α becoming involved in the activation of the NF-κB signaling pathway in HLEC. 

## 2. Materials and Methods

### 2.1. Reagents

Recombinant human TNF-α was obtained from Prospec-Tany TechnoGene Ltd. (Rehovot, Israel). 3-(4,5-dimethylthiazol-2-yl)-2,5 diphenyltetrazolium bromide (MTT) was purchased from Sigma-Aldrich (St. Louis, MO, USA). Bovine serum albumin (BSA) was purchased from Roche (Mannheim, Germany). Primary antibody against CCR7 was obtained from Abcam plc. (Abcam, Cambridge, UK). CCL21 neutralization antibody was obtained from R&D Systems (Inc, Minneapolis, MN 55413 USA). Antibodies against IκBα, NF-κB (p65) (c-20) and β-actin were obtained from Santa Cruz Biotechnology (Santa Cruz, CA, USA). Antibodies for IKKα, p-IKKα (T23), p53, Bax, Bcl-2, active-anti-Caspase-3, glyceraldehyde-3-phosphate dehydrogenase (GAPDH) and Lamin A were obtained from Abclonal Technology Company Ltd. (Wuhan, China). Antibodies for p-IκBα (Ser32), Caspase-3 and α-Tubulin were obtained from Cell Signaling Technology, Inc. (Beverly, MA, USA). IRDye®800-conjugated secondary antibodies were from Rockland Inc. (Philadelphia, PA, USA). A nuclear/cytosol fractionation kit (KeyGEN, Nanjing, China) was used according to the manufacturer’s directions. Annexin V/PI Cell Apoptosis Detection Kit, RNA Isolater Total RNA Extraction Reagent, HiScript II 1st Strand cDNA Synthesis Kit, and Synergy Brands (SYBR) Green PCR Master Mix were purchased from Vazyme biotec (Nanjing, China). CCR7 siRNA and NF-κB siRNA were purchased from GenePharma (Shanghai, China), and transfection was performed using Lipofectamine 2000 reagent (Invitrogen, San Diego, CA, USA), according to the manufacturer’s instructions. A human CCL21 ELISA kit was obtained from Boster Biological Technology co.ltd. (Wuhan, China). 

### 2.2. Cell Culture

Human lung cancer cells A549 and H460, human lymphatic endothelial cell (HLEC), human breast cancer cells MCF-7 and MDA-MB-231, hepatoma carcinoma cell HepG2, and acute myeloid leukemia (AML) cells MyLa, T-cell lymphoma cells HuT-102 and HuT-78 were originally obtained from the Cell Bank of the Shanghai Institute of Cell Biology. The cells were cultured in Dulbecco’s modified Eagle’s medium (DMEM) medium (Gibco, Grand Island, NY, USA) containing 10% fetal bovine serum (FBS) (Gibco), 100 U/mL penicillin, and 100 μg/mL streptomycin, in a humidified 5% CO_2_ atmosphere at 37 °C.

### 2.3. MTT Assay

Cells were seeded into 96-well plates (Corning, New York, NY, USA) at a density of 1 × 104 cells/well in 100 μL fresh DMEM medium and allowed to attach overnight. Each group consisted of six parallel wells. Then the cells were exposed to different concentrations of TNF-α in a humidified 5% CO2 incubator at 37 °C. After 48 h, 20 μL/well of MTT (5 mg/mL) was added to the medium and the cells were incubated at 37 °C for 4 h. The supernatant was removed and 100 μL dimethyl sulfoxide (DMSO) was added to dissolve the precipitate of formazan crystals. The plates were shocked for 2 min, then absorbance was measured spectrophotometrically at 570 nm on an automated microtiter plate reader (EL800, BIO-TEK Instruments Inc., Winooski, VT, USA). All assays were performed in triplicate.

### 2.4. Cytokine Quantification by Enzyme-Linked Immunosorbent Assay (ELISA)

Cells were cultured on 24-well plates at a density of 4 × 104 cells/well, and then were treated with different concentrations of TNF-α for 48 h. The supernatant was collected and the concentration of CCL21 secretion in cell supernatants was measured by Human CCL21 ELISA Kit according to the manufacturer’s instructions. The experiments were repeated three times, medium without serum was used. 

### 2.5. Real-Time PCR Analysis

Cells were treated with different concentrations of TNF-α for 48 h. The mRNA levels of CCL21 was then determined with a method described previously [23]. Forward and reverse primers for targeted mRNA were designed and purchased from Sangon Biotech (Shanghai, China). The primer sets used for the PCR amplifications were as follows: 

CCL21

forward, 5′-GCTCTAGAATGGCTCAGTCACTGGCTCT-3′

reverse, 5′-ATATGCGGCCGCCTATGGCCCTTTAGGGGTCTGT-3′

GAPDH

forward, 5′-TGGGTGTGAACCATGAGAAG-3′

reverse, 5′-GCTAAGCAGTTGGTGGTGC-3′

### 2.6. Preparation of Cytosolic and Nuclear Protein Extracts

Cells were seeded in cell cultured flasks and were treated with different concentration of TNF-α (10, 20 and 40 ng/mL) for 48 h. Then the cells were collected and cytosolic and nuclear fractions were prepared using a nuclear-cytosol fractionation kit (KEYGEN BioTECH, Nanjing, China). Cells were washed with 1 mL of precooled PBS and then centrifuged in 4 °C at 3000 rpm for 5 min. The supernatant fraction was removed, and the sediment was resuspended by indicated volume of Buffer A. The samples were vortexed for 15 s at maximum speed, then placed on ice for 15 min. Then 11 uL Buffer B was added, and the samples were vortexed 5 s and placed on ice for 1 min, then the samples were vortexed 5 s and centrifuged at 14,000 rpm for 10 min at 4 °C. The supernatant fraction was collected and stored at −80 °C or used immediately. The sediment containing nuclear proteins was resuspended by an indicated volume of Buffer C. The samples were vortexed for 15 s and then placed on ice for 40 min. The samples were vortexed every 10 min. Then the samples were centrifuged at 14,000 rpm for 15 min at 4 °C. The supernatant fractions were collected. The concentrations of the cytosolic and nuclear proteins were measured by Bicinchoninic acid (BCA) assay kit (Pierce, Rockford, IL, USA) with a Varioskan multimode microplate spectrophotometer (Thermo, Waltham, MA, USA). The cytosolic and nuclear fractions were subjected to Western blot analysis.

### 2.7. Western Blot

Cells were collected and lysed in protein lysis buffer (50 mM Tris-Cl, pH 7.6, 150 mM NaCl, 1 mM Ethylene Diamine Tetraacetic Acid (EDTA), 1% (m/v) Nonidet P-40 (NP-40), 0.2 mM phenylmethanesulfonyl fluoride (PMSF), 0.1 mM NaF and 1.0 mM dithiothreitol). The lysates were clarified by centrifugation at 4 °C for 20 min at 14,000 rpm. The concentration of protein in the supernatants was measured using a BCA assay kit. An equal amount of protein lysate (50 μg) was placed on a sodium dodecyl sulfate-polyacrylamide gel electrophoresis (SDS-PAGE) system for 2 h at 100 V, followed by transfer of the protein to an Nitrocellulose filter (NC) membrane (Millipore, Boston, MA, USA). The blots were blocked with 3% BSA in PBS at 37 °C for 1 h and were incubated with specific antibodies against indicated primary antibodies overnight at 4 °C. The membranes were washed three times with PBS containing 1% Tween-20, and probed with IRDyeTM 800-conjugated secondary antibody for 1 h at 37 °C. Blots were reacted with enhanced chemiluminescence (ECL) reagent (Bio-Rad, Hercules, CA, USA) and signals were detected by Amersham Imager 600 (GE, Piscataway, NJ, USA). 

### 2.8. Cell Apoptosis

Cell apoptosis was evaluated using an AnnexinV-FITC/PI Apoptosis Detection Kit (Vazyme, city, State, Country) according to the manufacturer’s instructions. In brief, 1 × 10^6^ A549 cells were incubated with the supernatant from different concentration of TNF-α (10, 20 and 40 ng/mL)-pretreated HLEC cells. After 48 h, the cells were harvested and washed with PBS and Binding Buffer. Next, they were incubated with 5 µL of Annexin V and 5 µL of Propidium Iodide (PI) in Binding Buffer for 10 min at room temperature protected from light. Cellular fluorescence was measured by flow cytometric analysis using a Fluorescence Activating Cell Sorter (FACS) Calibur BD CellQuest Pro system (San Jose, CA, USA).

### 2.9. Transient Transfection with siRNA

Cells were plated in six-well plates with fresh DMEM medium. The siRNA transfection was performed using Lipofectamine 2000 reagent according to the manufacturer’s instructions.

### 2.10. Cell Adhesion Assay

HLEC was treated with different concentrations of TNF-α (10, 20 and 40 ng/mL) for 48 h, the supernatant was removed, and cells were re-cultured with fresh DMEM medium for 48 h. Then A549 cells were treated with the supernatant of HLEC for 48 h, and then the cells were collected. A549 cells were washed by PBS and resuspended by serum free DMEM to 2 × 104 cells /100 uL. The 96-well plates were coated with 50 uL Matrigel (Corning, NY, USA, diluted 1:1 in DMEM medium) at 37 °C for 2 h and then 100 uL 2% BSA solution was added into every well at 4 °C for 4 h. The A549 cells were seeded on the 96-well plates and then placed in the incubator for 1 h at 37 °C. Then the medium was discarded and washed by PBS. Then 20 uL MTT was added into the wells and incubated at 37 °C for 4 h, the supernatant was discarded, and then every well to was added 100 uL DMSO, the absorbance was measured at 570 nm.

### 2.11. Cell Invasion Assay 

The invasion of A549 cells was analyzed by Transwell invasion assay. An amount of 1 × 105 HLEC were seeded into 24-well plates, and then treated with different concentrations of TNF-α (10, 20 and 40 ng/mL) for 48 h. The supernatant was removed, and cells were re-cultured by fresh DMEM medium. The Transwell chambers (12 mm in diameter, 8 μm poresize, Millipore, Billerica, MA, USA) were coated with 100 uL Matrigel (Corning, NY, USA, diluted 1:8 in DMEM medium) at 37 °C and 5% CO^2^ 30 min. Then the Transwell chambers were put in the 24-well plates containing the HLEC. An amount of 2 × 105 A549 cells were suspended by 400 uL serum free medium and then added into the upper transwell chamber. An amount of 600 uL fresh medium was added to the bottom chamber. Followed cells were placed at 37 °C for 48 h. Non-invaded cells were removed with cotton swabs, and the chamber was fixed by 100% methyl alcohol for 10 min and stained by hematoxylin and eosin. Invaded cells were counted under microscope. Then, they were quantified by manual counting and three randomly chosen fields were analyzed for each group.

### 2.12. Statistical Analysis

All data were obtained from at least three independent experiments and all experiments were performed in a parallel manner. The data in different experimental groups were expressed as the mean ± Standard Error of Mean (S.E.M). Differences of multiple group comparisons were tested with one-way analysis of variance (ANOVA) followed by the Bonferroni post-hoc test. Comparisons between two groups were analyzed using two-tailed Student’s *t*-tests. The significance of differences is indicated at * *p* < 0.05 and ** *p* < 0.01. 

## 3. Results

### 3.1. CCR7 is Overexpressed in Metastatic Lung Cancer

**Figure 1:**
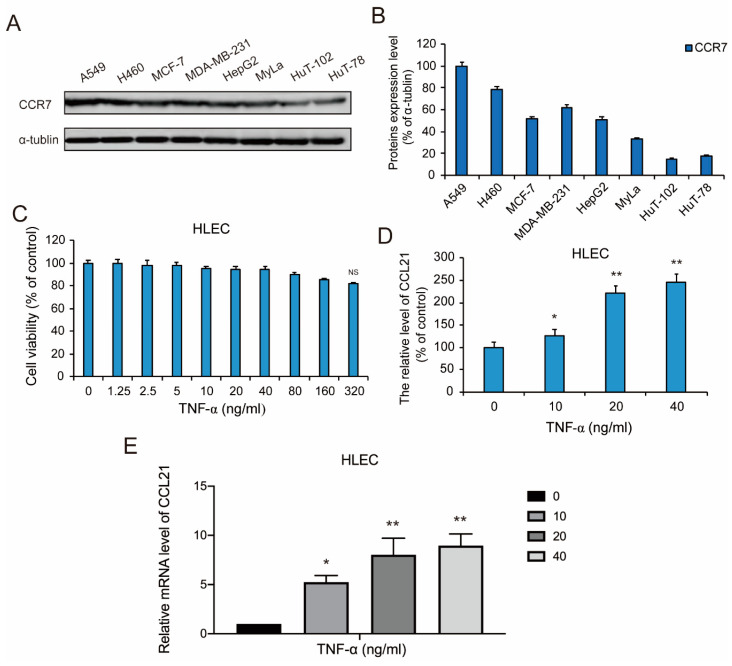
CCR7 is overexpressed in A549 non-small cell lung cancer (NSCLC) cells and TNF-α induced the secretion of CCL21 in human lymphatic endothelial cells (HLEC). (**A,B**) The expression levels of CCR7 protein in NSCLC cells A549 and H460, human breast cancer cells MCF-7 and MDA-MB-231, hepatoma carcinoma cell HepG2, acute myeloid leukemia (AML) cells MyLa, T-cell lymphoma cells HuT-102 and HuT-78. Western blotting was performed to detect the expression of the listed proteins, using α-tubulin as loading controls. Data represent the mean ± S.E.M. from three independent experiments. (**C**) HLEC in logarithmic growth phase were incubated in 96-well plates with 1 × 10^4^ cells in 100 μL DMEM culture medium, then were treated with 100 μL various concentrations (0–320 ng/mL) of TNF-α for 48 h, respectively. The cell viability effect of TNF-α on the cell lines was determined using an MTT assay. Data were shown as mean ± S.D. (*n* = 6). (**D**) After HLEC was treated with or without TNF-α (10, 20, and 40 ng/mL) for the 48-h time point, Then the TNF-α was removed and replaced with fresh medium to continue culturing for 48 h. ELISA analysis of CCL21 secretion in HLEC was performed. Data represent the mean ± S.E.M. from three independent experiments. (**E**) qRT-PCR analysis of gene products associated with cellular CCL21. RNA was prepared from HLEC treated with or without TNF-α (10, 20, and 40 ng/mL) for the 48-h time point and qRT-PCR was performed as described in Materials and Methods. Representative histograms of three independent experiments are shown. Data represent the mean ± S.E.M. from three independent experiments (* *p* < 0.05 and ** *p* < 0.01).

We collected a series of cancer cells, including NSCLC cells A549 and H460, human breast cancer cells MCF-7 and MDA-MB-231, hepatoma carcinoma cell HepG2, and acute myeloid leukemia (AML) cells MyLa, T-cell lymphoma cells HuT-102 and HuT-78. Western blotting results showed that the expression of CCR7 in NSCLC A549 and H460 cells is higher than other cell lines (Figure 1A; Figure 1B). It is reported that lung adenocarcinoma tumor cells highly expressed the chemokine receptor CCR7, and tumor cells with positive expression of CCR7 preferentially transferred to the CCR7 ligand CCL21-enriched lymphoid organs, which provides a basis for preferential metastasis of tumor cells to specific sites. These data indicated that high expression of CCR7 may be an important cause of the metastasis of NSCLC cells. Therefore, we chose the A549 cells and H460 cells to investigate the effect of the CCR7–CCL21 axis on lymphatic metastasis. 

### 3.2. TNF-α Induced the Secretion of CCL21 in HLEC

TNF-α can regulate the tumor microenvironment balance by promoting chemokine secretion. The co-culture system of primary lymphatic endothelial cells and lung adenocarcinoma cells found that TNF-α significantly increased the concentration of CCL21 in lymphatic endothelial cell culture medium and could promote the invasion and metastasis of lung adenocarcinoma cells. Therefore, in order to investigate the effect of TNF-α on the secretion of CCL21 in HLEC, we next first measured the cell growth effect of TNF-α on HLEC. An MTT assay found that the treatment with TNF-α (1.25–320 ng/mL) for 48 h did not show significant inhibition of cell viability of HLEC (Figure 1C). Further, we selected three doses of TNF-α (10, 20 and 40 ng/mL) to explore the production of CCL21. These concentrations were also applied to all subsequent experiments. ELISA results demonstrated that the secretion of CCL21 was increased in HLEC cell in a dose-dependent manner after TNF-α was removed for 48 h (Figure 1D). Consistently, RT-PCR results showed that the mRNA expression levels of CCL21 were significantly increased by TNF-α in HLEC for 48 h (Figure 1E). These data suggested that TNF-α could induce the secretion of CCL21 in HLEC at the level of transcription.

### 3.3. TNF-α Activated NF-κB Signaling Pathway in HLEC

**Figure 2:**
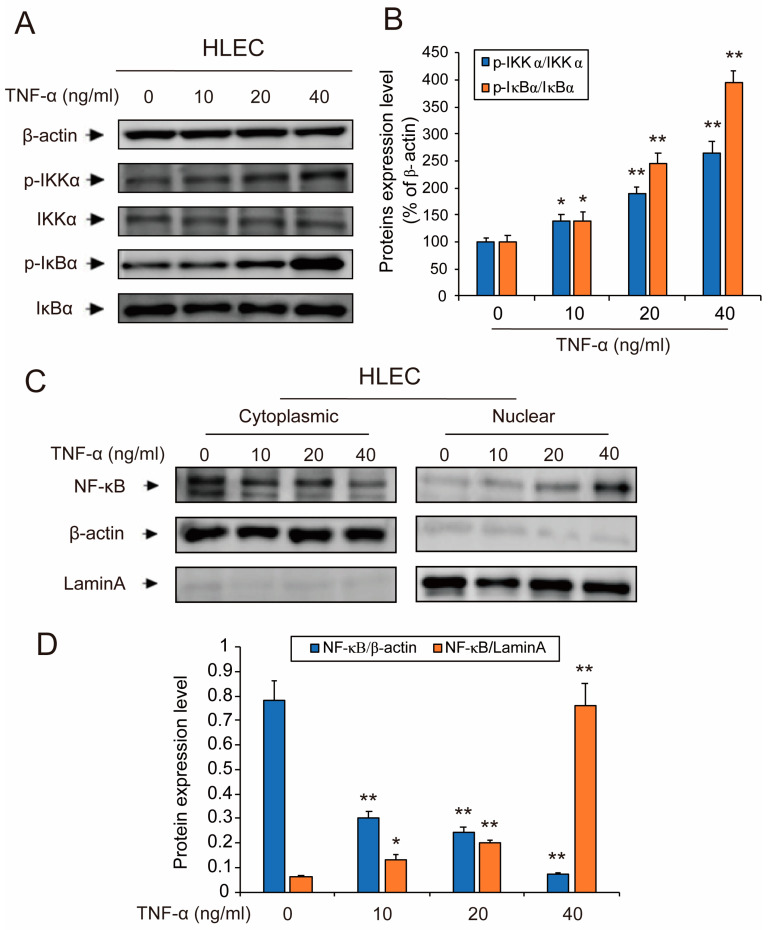
TNF-α activated the NF-κB signaling pathway in HLEC. (**A**,**B**) Changes in the expression of IKKα, p-IKKα, IκBα, and p-IκBα proteins following treatment with TNF-α (10, 20, and 40 ng/mL) for the 48-h time point in HLEC. Western blotting was performed to detect expression changes of the listed proteins, using β-actin as loading controls. Data represent the mean ± S.E.M. from three independent experiments. (**C**,**D**) After HLEC was treated with TNF-α (10, 20, and 40 ng/mL) for the 48-h time point, the cytoplasmic/membrane and nuclear fractions of the HLEC were analyzed by Western blotting for the NF-κB nuclear translocation, with β-actin and Lamin A as cytoplasmic and nuclear loading controls, respectively. Data represent the mean ± S.E.M. from three independent experiments. Asterisks denote statistically significant (* *p* < 0.05 and ** *p* < 0.01) differences compared with controls by one-way ANOVA.

To further elucidate the mechanism of TNF-α-induced secretion of CCL21 in HLEC, we next investigated the effect of TNF-α on NF-κB pathway activation. Western blotting results revealed that TNF-α could significantly activate the NF-κB pathway in HLEC after treatment for 48 h. Both p-IKKα and p-IκBα were significantly up-regulated by TNF-α in a concentration-dependent manner (Figure 2A,B). Next, nuclear and cytoplasm/membrane proteins of HLEC were fractionated in the presence of TNF-α for 48 h. Similarly, we observed that TNF-α treatment promoted the nuclear translocation of NF-κB, demonstrating the expected effects of TNF-α on NF-κB pathway activation of HLEC (Figure 2C,D).

### 3.4. TNF-α-Induced Secretion of CCL21 Involving the NF-κB Signaling Pathway in HLEC

**Figure 3:**
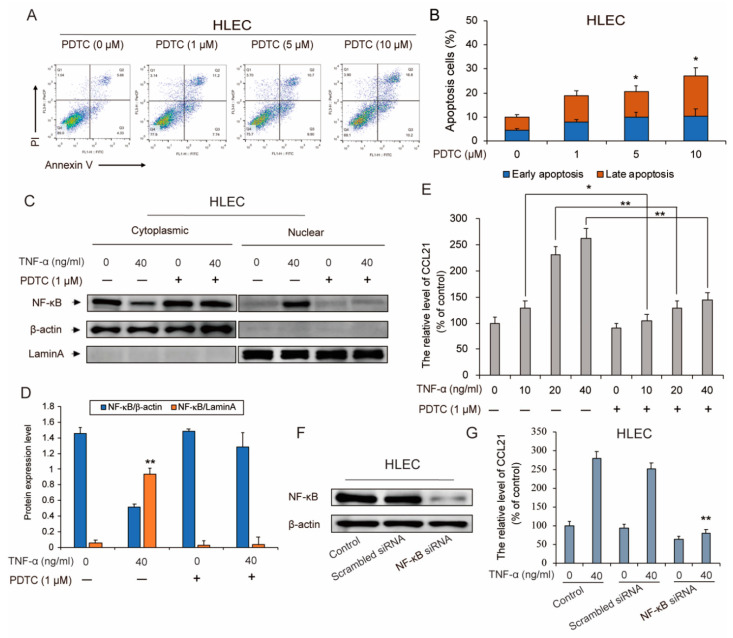
TNF-α-induced secretion of CCL21 involving the NF-κB signaling pathway. (**A**,**B**) After HLEC were treated with PDTC (1, 5, and 10 µM) for the 48-h time point, cells were incubated with Annexin V-FITC and PI as described in Materials and Methods to assess the cell apoptosis. Annexin V-and/or PI-positive cells were counted under a fluorescence microscope and expressed as a percentage of the total cell number. Results were analyzed by flow cytometry. Early- and Late-apoptosis ratios of HLEC are shown; columns represent means of three different experiments; bars represent standard errors. (**C**,**D**) Afterwards, HLEC was pre-treated with or without pyrrolidinedithiocarbamate (PDTC) (1 µM) for the indicated time periods, and then treated with or without TNF-α (40 ng/mL) for the 48 h-time point. The cytoplasmic/membrane and nuclear fractions of the HLEC were analyzed by Western blotting for the NF-κB nuclear translocation, with β-actin and Lamin A as cytoplasmic and nuclear loading controls, respectively. Data represent the mean ± S.E.M. from three independent experiments. Asterisks denote statistically significant (* *p* < 0.05 and ** *p* < 0.01) differences compared with controls by one-way ANOVA. (**E**) ELISA analysis of the secretion of CCL21 following pre-treatment with PDTC (1 µM) for the indicated time periods, and then treated with or without TNF-α (10, 20, and 40 ng/mL) for the 48-h time point in HLEC. Data represent means ± S.E.M. from three independent experiments. Asterisks denote statistically significant (* *p* < 0.05 and ** *p* < 0.01) differences compared with controls by one-way ANOVA. (**F**,**G**) HLEC were transfected with 10 nM Scrambled siRNA or NF-κB siRNA, and the expression of NF-κB was determined by Western blotting. The secretion of CCL21 in TNF-α (40 ng/mL)-treated HLEC which were transfected with Scrambled siRNA or NF-κB siRNA, was evaluated by ELISA analysis assay.

As shown in Figure 1; Figure 2, the results demonstrated that TNF-α increased the secretion of CCL21, meanwhile the NF-κB pathway was activated in HLEC. Previous study has demonstrated that the NF-κB pathway is involved with the regulation of CCL21. Therefore, we examined whether the TNF-α-induced CCL21 secretion effect of HLEC is dependent on the NF-κB pathway. Then, we chose the pyrrolidinedithiocarbamate (PDTC), an inhibitor of the NF-κB signaling pathway, to test the effect of NF-κB pathway activation on production of CCL21. We first measured the apoptosis effect of PDTC on HLEC. Annexin V/PI double staining assay results demonstrated that 5 µM PDTC treatment began to induce a more obvious apoptotic effect in HLEC (the ratio of apoptosis cells > 20%) for 48 h (Figure 3A,B). Therefore, we selected a dose of 1 µM PDTC that could not induce a significant apoptotic effect in HLEC to carry out the next study. Moreover, to further confirm the effect of the NF-κB pathway, HLEC were pretreated with PDTC, an inhibitor of the NF-κB signaling pathway, then TNF-α was added. Next, nuclear and cytoplasm/membrane proteins of HLEC were fractionated. Results showed that the TNF-α-induced nuclear translocation of NF-κB was significantly inhibited (Figure 3C,D). Moreover, the ELISA assay demonstrated that the production of CCL21 in HLEC was markedly inhibited for 48 h (Figure 3E).

Similarly, we also used NF-κB siRNA to knock down the expression of NF-κB in HLEC to confirm the effect of NF-κB pathway activation on CCL21 secretion. The expression of NF-κB was obviously decreased by NF-κB siRNA (Figure 3F). Then, the results of ELISA analysis demonstrated that the silencing of NF-κB expression led to the inhibition of CCL21 secretion (Figure 3G). In conclusion, it suggested that TNFα-induced secretion of CCL21 in HLEC is involved in the NF-κB signaling pathway activation.

### 3.5. Co-Culture of HLEC and NSCLC Cells Promoted the Invasiveness and Migration of CCR7-Overexpressed NSCLC Cells

**Figure 4:**
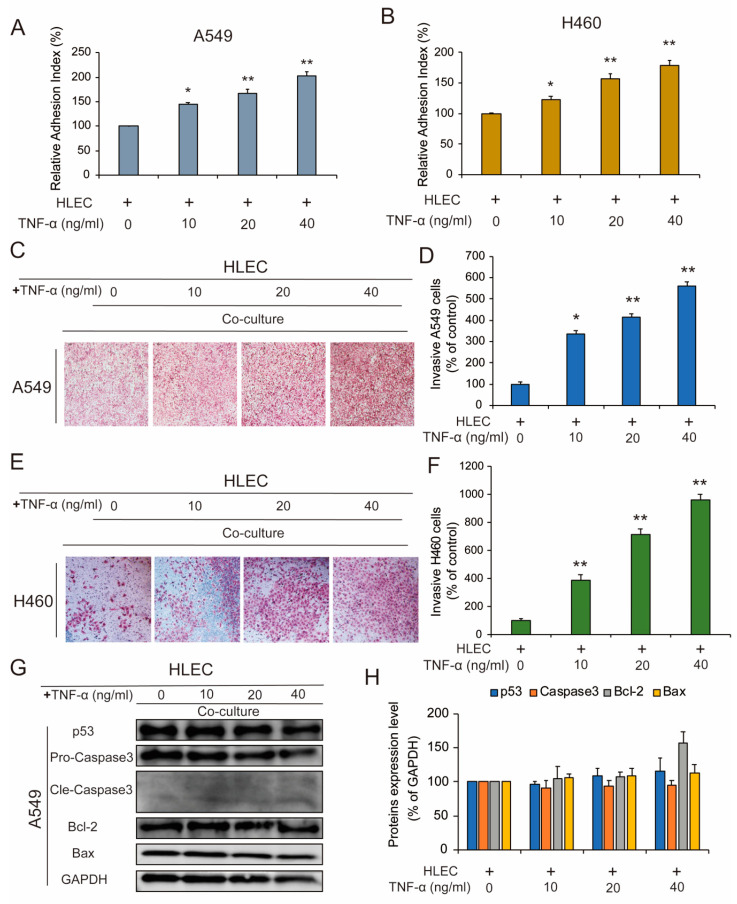
Co-culture of HLEC and A549 cells promoted the lymphatic metastasis of CCR7-overexpressed NSCLC cells. HLEC were exposed to different concentrations of TNF-α (10, 20, and 40 ng/mL) for 48 h. Then the TNF-α was removed and replaced with fresh medium to continue culturing for 48 h. Next, A549 or H460 cells were co-cultured with the above HLEC for 48 h. (**A,B**) A549 cells or H460 cells were collected and 100 μL cell suspension (2 × 10^5^ cells/mL) was added to the 96 wells which are pre-coated with matrigel. After incubating for 1 h, adherent cells were determined by MTT assay. Data represent the mean ± S.E.M. from three independent experiments. Asterisks denote statistically significant (* *p* < 0.05 and ** *p* < 0.01) differences compared with controls by one-way ANOVA. (**C,D**) The invasive ability of A549 cells was evaluated by a matrigel-coated transwell invasion assay (image magnification: 200×). Each experiment was performed at least three times. Data are presented as mean ± S.D. * *p* < 0.05 compared with the control group; ** *p* < 0.01 compared with the control group. (**E,F**) The invasive ability of H460 cells was evaluated by a matrigel-coated transwell invasion assay (image magnification: 200×). Each experiment was performed at least three times. Data are presented as means ± S.D. * *p* < 0.05 compared with the control group; ** *p* < 0.01 compared with the control group. (**G,H**) Changes in the expression of some cellular apoptosis controlling proteins (p53, Caspase-3, Bcl-2 and Bax) in A549 cells following co-culture with TNF-α (10, 20, and 40 ng/mL)-pretreated HLEC. Western blotting was performed to detect expression changes of the listed proteins, using glyceraldehyde-3-phosphate dehydrogenase (GAPDH) as loading controls. Data represent the mean ± S.E.M. from three independent experiments.

In order to investigate the metastasis promotion effect of CCL21 on NSCLC cells, we next used TNF-α to induce the secretion of CCL21 of HLEC; after 48 h, TNF-α was removed and replaced with normal medium. After 48 h of culture, NSCLC cells and HLEC were then co-cultured for 48 h. Cancer cell adhesion to basement membranes is important for tumor invasion since it is a key step in proteinase-dependent cell locomotion. The results of cell attachment assay showed that the adhesive capabilities of A549 cells and H460 cells were increased after co-culture with HLEC (Figure 4A,B). We next examined the effect of co-culture of A549 cells and TNF-α-pretreated HLEC on the invasion and migration of A549 cells. We found that cells in the control group were slightly invaded through the matrigel, whereas the ability was enhanced by co-culture with TNF-α-pretreated HLEC. Results showed that TNF-α-pretreated HLEC could promote the invasion of A549 cells in a concentration dependent manner, and the 40 ng/mL TNF-α-treated HLEC could cause about a 460% increase in the mobility of A549 cells (Figure 4C,D). Then we performed the same assay in H460 cells, we got a similar result as in A549 cells (Figure 4E,F). In addition, to further prove that the co-culture system of A549 cells and TNF-α-pretreated HLEC did not cause apoptosis in A549 cells, we therefore examined the expression of four apoptosis-related proteins in A549 cells after co-culture for 48 h. The analysis results showed that there was no significant expression change in the expression of apoptotic proteins, including the p53, Caspase-3, Cle-Caspase3, Bcl-2 and Bax, when compared with that in the control group. The data proved that the co-culture system prevented the cell apoptosis effect of A549 cells while inducing the lymphatic metastasis of the cells (Figure 4G,H).

### 3.6. HLEC Cell Co-Culture-Induced Metastasis and Invasion of A549 Cells is CCL21 and CCR7 Dependent

**Figure 5:**
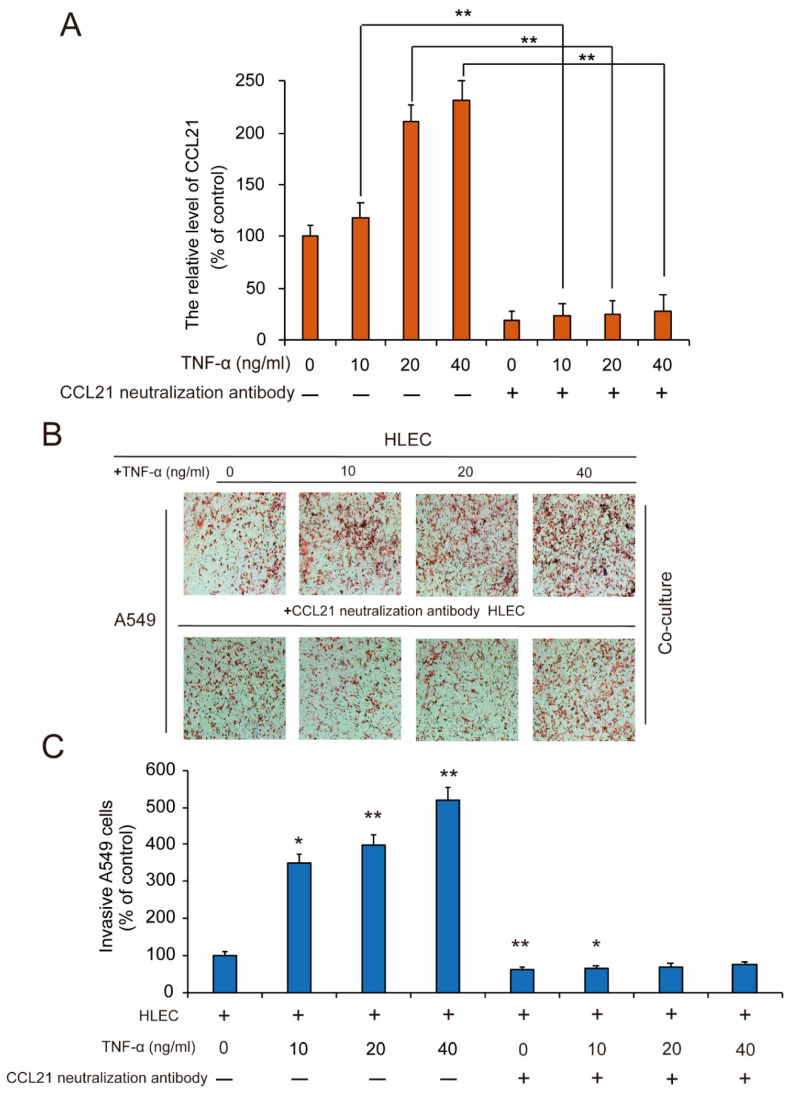
HLEC co-culture-induced metastasis and invasion of A549 cells is CCL21 dependent. HLEC were pretreated with or without the indicated concentrations of CCL21 neutralization antibody, and then exposed to different concentrations of TNF-α (10, 20, and 40 ng/mL) for 48 h. (**A**) Confirmation of the neutralized efficiency of CCL21 secretion was detected by ELISA analysis in HLEC. Data represent the mean ± S.E.M. from three independent experiments. Asterisks denote statistically significant (* *p* < 0.05 and ** *p* < 0.01) differences compared with controls by one-way ANOVA. (**B,C**) Afterwards, HLEC were exposed to the CCL21 neutralization antibody and different concentrations of TNF-α (10, 20, and 40 ng/mL) for 48 h, then the CCL21 neutralization antibody and TNF-α were removed and replaced with fresh medium to continue culturing for 48 h. Next, A549 cells were co-cultured with the above HLEC for 48 h. The invasive ability was evaluated by a matrigel-coated transwell invasion assay (image magnification: 200 ×). Each experiment was performed at least three times. Data are presented as means ± S.D. * *p* < 0.05 compared with the control group; ** *p* < 0.01 compared with the control group.

Since the treatment of TNF-α in HLEC could induce the production of CCL21 efficiently and CCL21 was used to activate CCR7, therefore, we speculated that co-culture of A549 cell and TNF-α-treated HLEC can significantly promote the migration of A549 cells, which may be related to the secretion of CCL21. Therefore, we next used CCL21 neutralizing antibody to verify this conjecture. First, we tested the efficiency of CCL21 neutralizing antibody, and found that after the addition of CCL21 neutralizing antibody, the secretion of CCL21 induced by TNF-α in HLEC was significantly suppressed (Figure 5A). Furthermore, the results of the Transwell experiment showed that after adding CCL21 neutralizing antibodies and co-culturing with TNF-treated HLEC, the migration capacity of A549 cells was significantly inhibited (Figure 5B,C). It suggested that the CCL21 secreted by TNF-α-treated HLEC is involved in the process of cancer metastasis. Meanwhile, the results indicated that the CCR7–CCL21 axis prevented the cell apoptosis and promoted the cell migration of A549 cells in the co-culture system of A549 cells and HLEC.

**Figure 6:**
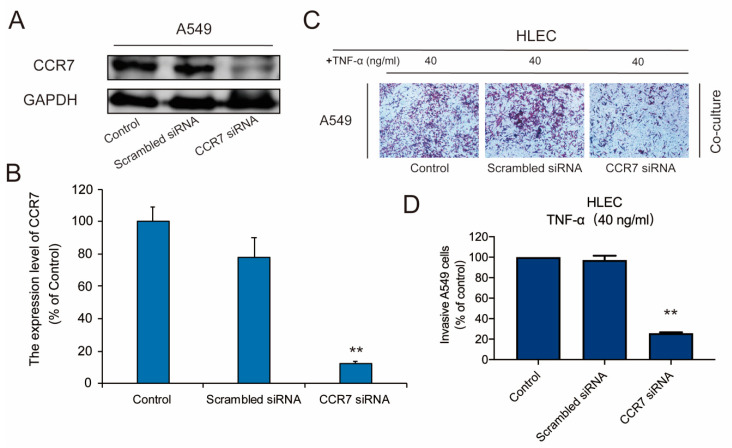
HLEC co-culture-induced metastasis and invasion of A549 cells is CCR7 dependent. A549 cells were transfected with Scrambled siRNA and CCR7 siRNA, and then co-cultured with HLEC which were pretreated with 40 ng/mL TNF -α, for 48 h. (**A**,**B**) A549 cells were transfected with 10 nM Scrambled siRNA or CCR7 siRNA, and the expression of CCR7 was determined by Western blotting. Data represent mean ± S.E.M. from three independent experiments. Asterisks denote statistically significant (* *p* < 0.05 and ** *p* < 0.01) differences compared with controls by one-way ANOVA. (**C**,**D**) The invasive ability of A549 cells which were transfected with Scrambled siRNA or CCR7 siRNA, was evaluated by a matrigel-coated transwell invasion assay (image magnification: 200 ×). Each experiment was performed at least three times. Data are presented as mean ± S.D. ** *p* < 0.01 compared with the control group.

In addition, we also used CCR7 siRNA to knock down the expression of CCR7 in A549 cells to confirm the effective role of the CCR7/CCL21 axis in NSCLC cells metastasis. The expression of CCR7 was obviously reduced by CCR7 siRNA (Figure 6A,B). Then, the results of Transwell experiment demonstrated that the silencing of CCR7 expression led to the weaker metastatic propensity of A549 cells (Figure 6C,D). It suggested that the expression of CCR7 is necessary for the metastasis of A549 cells in the co-culture system.

## 4. Discussion

Lung cancer is currently the leading cause of cancer-related deaths worldwide. Non-small cell lung cancer (NSCLC) is responsible for nearly 85 percent of all lung cancer cases. Though targeted therapy and immunotherapy have made great progress, there is still a need to uncover other potential mechanisms of progression. Comprehensive treatments such as radical surgery, chemotherapy, radiotherapy, and targeted therapy have not yet achieved satisfactory results. Metastasis and recurrence are the main causes of death of patients with NSCLC [8]. At present, the specific mechanism of tumor metastasis is not clear, and the widely recognized factors affecting tumor metastasis include the formation of malignant metastasis phenotypes of tumor, the lack of adhesion factors on the surface of tumor cells, the role of extracellular matrix, the chemotaxis of tumor cells, and tumor angiogenesis and lymphatic vessels production [24,25]. Tumor metastasis is a complex sequential process that requires the involvement of a variety of factors and cellular reactions, including random or specific capture of the target tissue micro-vasculature, leaving the vein, growing, and immersing into the tissue organs until metastasis is formed [26]. Due to the lack of specific lymphocytic endothelial cell (LEC) markers, the research of the mechanism of tumor lymphatic metastasis has progressed extremely slowly, and lymphatic tubes have long been mistakenly perceived as passive during tumor metastasis. In recent years, with the discovery of a variety of specific LECs markers, it has been revealed that the lymphatic tube not only provides an anatomical basis for tumor metastasis, but also actively participates in the process of tumor metastasis through self-remodeling. Organs such as the brain, bone, liver and adrenal organ are the most vulnerable metastatic lesions of lung adenocarcinoma, suggesting that lung cancer metastasis has the characteristics of non-randomness and organ selectivity. However, a prominent problem needs to be solved, namely, how tumor cells recognize and enter the lymphatic tube to form a metastatic lesion to distant organs. Lymphatic metastasis is the initial stage of the tumor spreading outward; therefore, it is particularly important to study how to regulate this process to stifle tumor metastasis in the bud.

Many tumor cells can specifically express certain chemokine or chemokine receptors, and previous investigation revealed that the chemokine and its receptors are closely related to tumor invasion and metastasis. The chemokine has biased expression in some organs or tissues and, when combined with the tumor cell surface receptor, it can induce the directional migration of tumor cells’ inverse concentration gradient of chemokine, and this model of “chemokine-receptor” may be the mechanism of specific organ metastasis of certain solid tumors [27]. NSCLC cells express the C-C chemokine receptor type 7 (CCR7), and CCR7 expression-positive tumor cells are preferred to their ligand-enriched lymphatic organs, providing a basis for the priority transfer of tumor cells to specific sites [28]. Moreover, CCR7 activation has been demonstrated to mediate the carcinogenesis and progression of tumor [29]. In this study, we investigated the relation between CCR7 expression and the cell metastasis in A549 cells. The results revealed that the high expression of CCR7 in NSCLC A549 cells is involved in the metastasis. Moreover, chemokine ligand 21 (CCL21), a ligand of CCR7, was specifically used to upregulate and activate CCR7. CCL21 is highly expressed in lymph nodes and lymphatic endothelium. Moreover, tumor necrosis factor (TNF-α) is common in the microenvironment of human lymphatic endothelial cells (HLEC). TNF-α could assist some cells to produce several cytokines in the microenvironment. It has been confirmed that the chemokines CXCL8, CXCL12, CCL2 and CCL27 are the downstream regulatory targets of TNF-α [30,31,32,33]. However, the regulation effect of TNF-α on CCL21 has not been reported. Here, the results of a co-culture system of A549 cells and TNF-α-pretreated HLEC showed that A549 cells could undergo lymphocytic metastasis. In addition, the reports showed that the interaction of CCR7 and CCL21 is involved in various physiological processes including the proliferation and metastasis of different types of tumor cells, including breast, pancreatic and lung cancer. CCR7/CCL21 can promote the invasion and metastasis ability of B-cell chronic lymphocytic leukemia cells by activating phosphoinositide-3 kinase and the Rho effector molecule Rho-associated coiled-coil forming protein kinases (ROCK) [34]. Expression of CCR7, CCL21 and CCL19 play vital roles in the metastasis of CLL cells from the high endothelial venule to the lymph nodes [35]. In gastric cancer, the high expression of CCR7 and CCL21 can lead to the preferential metastasis of gastric cancer cells to lymphatic vessels [36]. CCR7 expression plays a key role in the ability of tumor cells to invade and metastasize in SW620 human colon cancer cell mice xenografted model [37]. CCR7 combined with the ligand can promote the metastasis of lung cancer cells by up-regulating the expression of sp1 [38]. The data revealed that the CCR7–CCL21 axis is one of the most common “chemokine-receptor” modes of action in the development and metastasis of multiple tumors. Therefore, CCR7–CCL21 may serve as a potential mechanism of lymphocytic metastasis or a therapeutic target for NSCLC. The study of CCL21/CCR7 will have important significance for the prevention and treatment of lung cancer metastasis.

To elucidate the underlying mechanisms involved in CCR7/CCL21-mediated cell metastasis, NF-κB signaling was further investigated in TNF-α-induced CCL21 production for HLEC. The results revealed that TNF-α markedly activated the NF-κB pathway and promoted the CCL21 production. Thus, PDTC, a specific antagonist of NF-κB, was used to inhibit NF-κB after TNF-α treatment. Thus, CCL21 was determined after NF-κB was antagonized and results revealed that the CCL21 secretion was significantly inhibited and the metastasis of A549 cell in the co-culture system was also decreased, which suggested that TNF-α-mediated CCL21 secretion in HLEC associated with NF-κB signaling and the lymphocytic metastasis of NSCLC cells. NF-κB signaling has been widely demonstrated to regulate cytokines [39,40]; similarly, the present data indicated that NF-κB was involved in TNF-α-mediated CCL21 production of HLEC. Thus, the research on whether the NF-κB signaling pathway activated by TNF-α can induce lymphatic metastasis with NSCLC will play a positive role in the research on the metastasis mechanism of lung cancer.

## 5. Conclusions

In conclusion, expression of CCR7 promotes cell proliferation and lymphatic metastasis in NSCLC cells. Moreover, further molecular mechanism studies via a co-culture system have shown that the metastasis event relies on TNF-α-induced secretion of CCL21 in HLEC, which may be associated with the activation of the NF-κB signaling pathway. This research provides a mechanistic insight in functioning of the CCR7/CCL21 axis and may contribute to develop the potential new target treatment in NSCLC with chemokine modulators.
